# Surgical management of nonbacterial thrombotic endocarditis in malignancy

**DOI:** 10.1186/s40792-017-0335-x

**Published:** 2017-05-05

**Authors:** Daisuke Kaneyuki, Kaoru Matsuura, Hideki Ueda, Hiroki Kohno, Michiyo Kanbe, Goro Matsumiya

**Affiliations:** 1 Department of Cardiovascular Surgery, Chiba University Hospital, 1-8-1 Inohana, Chuo-ku, Chiba-shi, Chiba 2608677 Japan; 2 Department of Diagnostic Pathology, Chiba University Hospital, 1-8-1 Inohana, Chuo-ku, Chiba-shi, Chiba Japan

**Keywords:** Nonbacterial thrombotic endocarditis, Infectious endocarditis, Valve diseases

## Abstract

**Background:**

Nonbacterial thrombotic endocarditis is commonly seen on heart valves in patients with malignant or collagen diseases. The natural prognosis of nonbacterial thrombotic endocarditis is reported to be poor due to underlying malignancy. Surgical indications and appropriate timing for surgery for nonbacterial thrombotic endocarditis and underlying malignancy have not been formally studied.

**Case presentation:**

The case was a 45-year-old woman who presented with a history of systemic embolization associated with occult malignancy. A preoperative transesophageal echocardiogram showed multiple mobile vegetations on the aortic and mitral valves. She underwent valve surgery to prevent recurrent embolization. Based on the histopathologic findings, she was diagnosed with nonbacterial thrombotic endocarditis. She subsequently underwent surgery for occult malignancy, which was diagnosed as endometrioid adenocarcinoma.

**Conclusions:**

Although surgical indications for nonbacterial thrombotic endocarditis remain unclear, valve replacement or repair and multidisciplinary treatment including surgical intervention are essential to prevent recurrent embolization in patients with nonbacterial thrombotic endocarditis associated with malignancy.

## Background

Nonbacterial thrombotic endocarditis (NBTE) is a disease characterized by the presence of vegetations on the cardiac valves, consisting of fibrin and platelet aggregates without inflammation or bacteria. NBTE is commonly seen on heart valves in patients with malignant or collagen diseases. Major treatment for NBTE includes anticoagulation and surgery. The natural prognosis of NBTE is reported to be poor due to underlying malignancy. We report a case with pathologically proven NBTE presenting with systemic thrombosis and associated with endometrioid adenocarcinoma, which was managed successfully with anticoagulation and valve surgery, followed by radical surgery for underlying malignancy.

## Case presentation

**Fig. 1:**
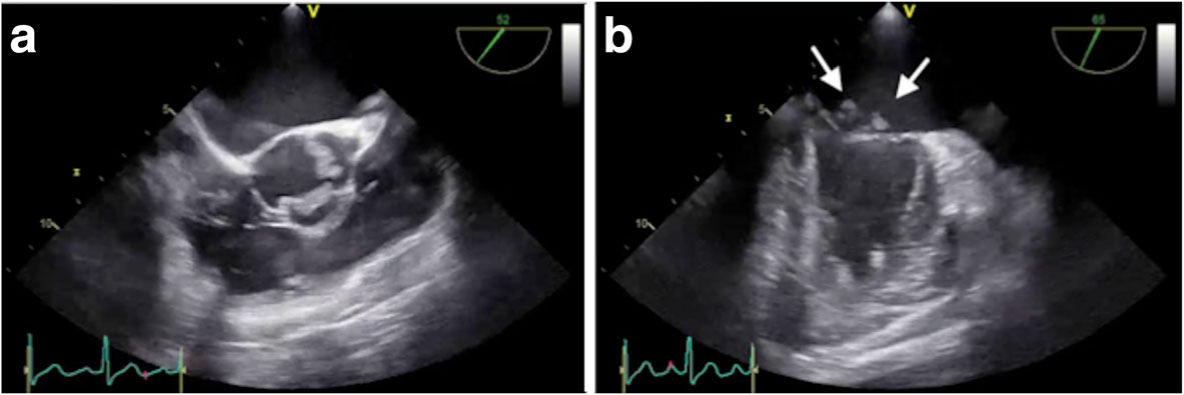
**a** Transesophageal echocardiogram shows structural change of left and right coronary cusps of aortic valve. **b** Vegetations on the mitral valve (*arrow*) in mitral commissural view. *LA* left atrium, *LV* left ventricle, *MV* mitral valve, *RA* right atrium, *TV* tricuspid valve, *RVOT* right ventricular outflow tract, *RV* right ventricle, *PV* pulmonary valve, *R* right coronary cusp, *L* left coronary cusp, *N* noncoronary cusp

A previously healthy 45-year-old woman presented with a 2-week history of tenderness in both lower legs and dizziness. She was suspected to have a cerebellar stroke, pulmonary emboli, and bilateral deep venous thrombosis on enhanced computed tomography at previous hospital. In addition, undiagnosed malignancy was suspected on the findings of abdominal computed tomography, which revealed a 4-cm enhanced irregular mass in sigmoid colon adjacent to the left ovary with poor demarcation. Then, she was transferred to our hospital for further evaluation and treatment. On presentation, her body temperature was 38.1 °C and did not have a heart murmur. Laboratory findings revealed elevated white blood cells counts, anemia, and markedly elevated CA 19-9. C-reactive protein was within normal range. There were no immunologic findings associated with lupus erythematosus, antiphospholipid syndrome, other collagen diseases, or hematologic disorders. The coagulation panel was within normal limits except for elevated D-dimer levels at 19.6 μg/ml. She was started on apixaban, which is a direct factor Xa inhibitor. Initially, general surgery team tried to perform colonoscopy; however, she had severe abdominal pain and fever soon after taking premedication for colonoscopy. Abdominal computed tomography showed occlusive ileus. Transverse colostomy was performed on the same day emergently for decompression. Then, she was referred to cardiology department to evaluate preoperative cardiac function before additional surgery for treatment and diagnosis. Preoperative transthoracic echocardiography (TTE) revealed mobile vegetations on the mitral and aortic valves. Transesophageal echocardiography (TEE) also showed that all three coronary cusps of the aortic valve had 7–8 mm vegetations. Mild aortic insufficiency was seen around the vegetations. Similarly, both anterior and posterior mitral leaflets were thickened and had vegetations. The anterior mitral leaflet also had a 15-mm mobile mass and a few string-like bodies. Mild mitral regurgitation was observed from the center of the valve orifice (Fig. 1).

Given the echocardiographic findings, infectious endocarditis was suspected. Although three separate blood cultures were negative, meropenem were started empirically. Head magnetic resonance imaging showed no infarction, bleeding, or infected arterial aneurysm. She underwent an emergent operation to prevent recurrent thrombotic emboli 4 weeks after chief complaint was initiated.

A median sternotomy was performed, and cardiopulmonary bypass was instituted via ascending aortic and bicaval cannulation. After aortic cross-clamping, myocardial protection was achieved by the retrograde administration of cold blood cardioplegia. Then, aortotomy was performed, and antegrade blood cardioplegia was selectively administered into the coronary ostia. Vegetations were observed on all three cusps of the aortic valve. Both left and right coronary cusps had been destroyed structurally, and valve replacement using a biological valve (Carpentier-Edwards PERIMOUNT Magna Ease, 21 mm) was required. Since we could not rule out infectious endocarditis at that time, we chose a biological valve instead of a mechanical valve to minimize the postoperative bleeding risk due to anticoagulation. Next, right-sided left atriotomy was performed. Similar to the aortic valve, vegetations were attached to the anterior and posterior leaflets of the mitral valve. We successfully performed valve repair by careful debridement and ring annuloplasty. Remaining of surface of mitral valve was ablated with electric cautery. Weaning from cardiopulmonary bypass was possible with inotropic support.

**Fig. 2:**
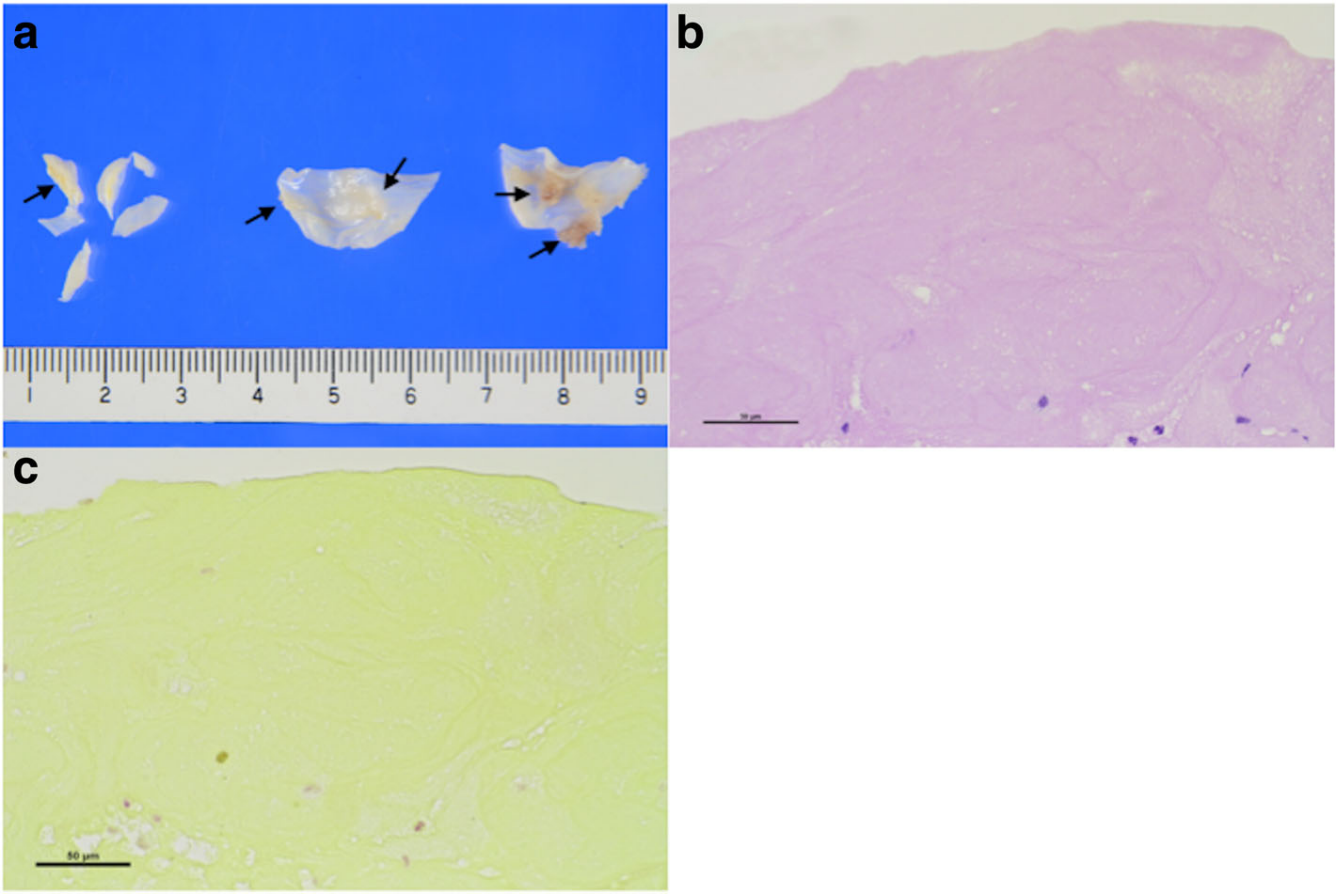
Histopathological findings of vegetation in aortic valve. **a** Gross photographs of valves with fibrotic thickening and small brown-colored vegetations (*arrows*). **b** Vegetation composed of fibrin (hematoxylin and eosin stain, magnification ×400). **c** No bacterial organisms seen (Gram stain, magnification ×400)

After the operation, anticoagulation therapy with warfarin was initiated. Follow-up TTE revealed no residual mitral regurgitation or vegetations on either the prosthetic aortic valve or native mitral valve. The pathology report revealed a few large brown-colored vegetations on both aortic and mitral valves that histopathologically consisted of eosinophilic fibrin. No bacterial organisms were detected microscopically (Fig. 2).

Based on these results, we suspected that the valve lesions were NBTE associated with occult malignancy. Three weeks after the cardiac operation, the general surgery team performed sigmoid colectomy and left salpingo-oophorectomy to remove the underlying malignancy causing thrombotic emboli. No mesenteric or hepatic metastases were observed. Histopathological examination revealed endometrioid adenocarcinoma in pelvic cavity and direct invasion in sigmoid colon. After 1-year follow-up, she has not experienced recurrent thromboembolic events on anticoagulation of warfarin and adjuvant chemotherapy for endometrioid adenocarcinoma.

### Discussion

NBTE was originally described by Ziegler in 1888 and has been reported to be associated with malignancy. Adenocarcinoma is the most common associated malignancy. Patients with adenocarcinoma reportedly have a fivefold higher risk of NBTE than patients with other histological types of malignant tumor [1]. Moreover, NBTE in the course of gynecological neoplasms (ovarian, cervical) is reported to have the highest risk of ischemic stroke [2].

Histological examination of the resected valve remains the gold standard for the diagnosis of NBTE. The vegetations of NBTE are friable, white or tan masses, usually situated along the lines of the valve closure, on the leaflets, which may be normal or previously damaged. They vary greatly in size from tiny lesions to large and exuberant masses. NBTE consists of degenerating platelets interwoven with strands of fibrin and forming a bland, featureless eosinophilic mass except for a few trapped leukocytes [3]. Differential diagnosis between NBTE and infectious blood culture-negative endocarditis can only be made histologically by identifying valvular inflammation, vegetation, and the organisms or other changes consistent with endocarditis.

Treatment of NBTE consists of systemic anticoagulation and therapy directed at treating the underlying malignancy or associated condition. Heparin is more effective than warfarin for hypercoagulability associated with malignancy [4–6]. However, Borowski et al. reported a case of recurrent embolism in the course of NBTE in spite of anticoagulation therapy [7], and other authors also have reported that treatment with heparin alone does not prevent further emboli [8]. This implies that anticoagulation therapy alone may not be sufficient to control hypercoagulability due to underlying malignancy. We cannot prevent recurrent embolization without removing the cancer in patients with NBTE associated with malignancy, because malignancy can still create a hypercoagulable state in spite of anticoagulation therapy. Eventually, this would result in recurrence of NBTE and thrombotic emboli.

Surgical indications and appropriate timing for surgery for NBTE have not been formally studied. In general, surgical indications for NBTE are the same as for infectious endocarditis and include uncontrollable heart failure or infection and mobile vegetations. In the present case, patient was relatively stable and TEE showed mobile vegetations which could cause stroke; emergent open-heart surgery was warranted. Although open-heart surgery might be waved because of systemic condition related to the malignancy, open-heart surgery should be performed as soon as possible when they are stable enough to underwent surgery and meet one of surgical indications listed above. Preservation of the valve might be possible in more cases of NBTE in which the valve is structurally and functionally normal after the vegetation is removed than that of bacterial infectious endocarditis.

Appropriate timing of surgery for underlying malignancy after the cardiac operation in NBTE has also not been reported. Our patient underwent surgery for underlying malignancy soon after histopathological examination confirmed a diagnosis of NBTE. We believe that surgical intervention should be performed as soon as possible once the diagnosis of NBTE is made after cardiac surgery to prevent recurrent vegetations and embolization.

## Conclusions

We experienced a case of NBTE with valvular destruction associated with underlying malignancy, which was successfully managed with valve surgery, anticoagulation therapy, and surgery for a malignant tumor. Although surgical indications for NBTE remain unclear, valve replacement or repair and multidisciplinary treatment including surgical intervention for underlying malignancy are essential to prevent recurrent embolization in patients with NBTE associated with malignancy.

## Abbreviations

NBTE: Nonbacterial thrombotic endocarditis
TEE: Transesophageal echocardiography
TTE: Transthoracic echocardiography
